# NEWS2 Is Superior to qSOFA in Detecting Sepsis with Organ Dysfunction in the Emergency Department

**DOI:** 10.3390/jcm8081128

**Published:** 2019-07-29

**Authors:** Lisa Mellhammar, Adam Linder, Jonas Tverring, Bertil Christensson, John H. Boyd, Parham Sendi, Per Åkesson, Fredrik Kahn

**Affiliations:** 1 Department of Clinical Sciences, Division of Infection Medicine, Lund University, 221 00 Lund, Sweden; 2 Department of Infectious Diseases, Skåne University Hospital, 22242 Lund, Sweden; 3 Department of Infectious Diseases, Helsingborg General Hospital, 25437 Helsingborg, Sweden; 4 Centre for Heart Lung Innovation, Division of Critical Care Medicine, St. Paul’s Hospital, University of British Columbia, Vancouver, BC V6Z 1Y6, Canada; 5 Institute for Infectious Diseases, University of Bern, 3001 Bern, Switzerland; 6 Department of Infectious Diseases and Hospital Epidemiology, University Hospital Basel, University Basel, 4031 Basel, Switzerland

**Keywords:** qSOFA, early warning score, NEWS2, sepsis, validation studies

## Abstract

Early administration of antibiotics is associated with better survival in sepsis, thus screening and early detection for sepsis is of clinical importance. Current risk stratification scores used for bedside detection of sepsis, for example Quick Sequential Organ Failure Assessment (qSOFA) and National Early Warning Score 2 (NEWS2), are primarily validated for death and intensive care. The primary aim of this study was to compare the diagnostic accuracy of qSOFA and NEWS2 for a composite outcome of sepsis with organ dysfunction, infection-related mortality within <72 h, or intensive care due to an infection. Retrospective analysis of data from two prospective, observational, multicentre, convenience trials of sepsis biomarkers at emergency departments were performed. Cohort A consisted of 526 patients with a diagnosed infection, 288 with the composite outcome. Cohort B consisted of 645 patients, of whom 269 had a diagnosed infection and 191 experienced the composite outcome. In Cohort A and B, NEWS2 had significantly higher area under receiver operating characteristic curve (AUC), 0.80 (95% CI 0.75–0.83) and 0.70 (95% CI 0.65–0.74), than qSOFA, AUC 0.70 (95% CI 0.66–0.75) and 0.62 (95% CI 0.57–0.67) *p* < 0.01 and, *p* = 0.02, respectively for the composite outcome. NEWS2 was superior to qSOFA for screening for sepsis with organ dysfunction, infection-related mortality or intensive care due to an infection both among infected patients and among undifferentiated patients at emergency departments.

## 1. Introduction

The incidence of sepsis is high (600/100 000 persons per year), with an in-hospital mortality of 16% [1,2]. Early administration of antibiotics is associated with better survival, thus screening and early detection for sepsis is of clinical importance [3]. Numerous scores have been devised both for detection and prognostication of sepsis. These include Systemic Inflammatory Response Syndrome (SIRS), Quick Sequential Organ Failure Assessment (qSOFA) and most recently, National Early Warning Score 2 (NEWS2) (Table S1) [4,5,6,7]. The use of these scores has also led to sepsis alert systems in which patients considered to be at high risk of critical illness are prioritized and treated according to sepsis bundles [8,9].

qSOFA was both derived and primarily validated using death and prolonged intensive care as outcomes [4,10]. However, qSOFA’s accuracy when used to predict sepsis is lower [11,12].

Although qSOFA was developed to identify those with risk of sepsis among infected patients, the authors recommended that positive qSOFA criteria should also prompt consideration of possible infection in patients not previously recognized as infected [13]. NEWS2 is a modified version of NEWS, which is commonly used in British hospitals for identifying patients at risk for deterioration [7].

For use as a screening tool amongst undifferentiated patients in the Emergency Department (ED), a score should be capable of discriminating sepsis from other competing diagnoses, as well as predicting the development of sepsis among infected patients [14]. Therefore, in this study we evaluated qSOFA and NEWS2 for their ability to detect sepsis, both among infected patients as well as in a broader cohort consisting of both infected and non-infected patients.

Although elevated serum lactate levels were originally considered for inclusion in qSOFA, lactate levels had little effect upon how patients were classified [13]. Heparin binding protein (HBP) is a granule protein, released by neutrophils in response to bacterial products [15,16,17]. HBP increases vascular leakage, correlates with progression to septic shock and has proven to be an early biomarker of severe sepsis in the ED [17,18,19,20].

The first aim of this study was to compare the diagnostic accuracy of qSOFA and NEWS2 for a composite outcome of sepsis with organ dysfunction (infection and an organ dysfunction defining severe sepsis according to sepsis-2), intensive care admission due to an infection and infection-related mortality within 72 h from enrolment. We went on to compare the predictive performance of qSOFA and NEWS2 for 30-day mortality, and last investigated whether including the biomarkers lactate and HBP improved performance for qSOFA and NEWS2

## 2. Methods

### 2.1. Patients

#### 2.1.1. Cohort A

Data were collected prospectively between 2011–2012 at EDs in an international, multicentre, convenience sampling, observational trial of sepsis biomarkers described elsewhere [21]. Adult patients were included at presentation if they had a suspected infection and at least one of SIRS criteria, or self-reported fever or chills. Only patients at the Swedish sites were included in this analysis. For details on including sites see Table S2. At enrolment, data on demography, comorbid conditions, medication and vital signs were recorded and blood drawn for plasma. Organ dysfunction, treatment, admission to intensive care, a diagnosis of infection and 30-days mortality was obtained from the hospital chart and the Swedish National death registry. Patients regarded as infected, as described below, were included. Patients discharged from the hospital, who did not die or require re-admission within 72 h were defined as not developing any new organ-dysfunction after discharge. The study was approved by the regional ethical board (2010/205).

#### 2.1.2. Cohort B

Data were collected prospectively between 2015–2016 at EDs in an international multicentre, observational trial of sepsis biomarkers [20]. For details on including sites see Table S2. At all sites except one (Helsingborg, Sweden) inclusion took place during fixed hours each weekday. During the time of inclusion (i.e., when study personal were present at the ED) there was no selection of patients. The inclusion at these sites (Lund, Vancouver and Bern) resembles a study with consecutive inclusion. Patients with one of the following parameters were included: respiratory rate >25 breaths per minute, heart rate >120 beats per minute, altered mental awareness, systolic blood pressure (SBP) below 100 mmHg, oxygen saturation (SaO_2_) <90%, or <93% if ongoing treatment with oxygen. At enrolment, data on demography, comorbid conditions, medication and vital signs were recorded and blood drawn for plasma. Organ dysfunction, treatment, admission to intensive care, a diagnosis of infection and 30-days mortality was obtained from the hospital chart and the Swedish National death registry. Both infected and non-infected patients were included in the analyses. Patients discharged from the hospital and not re-admitted within 72 h were defined as not having new organ-dysfunction after discharge. The study was approved by the regional ethical boards (Sweden 2014/41, Bern KEK 315/14, Vancouver H11-00505).

### 2.2. Definitions

We used the earlier sepsis-2 definition for organ dysfunction since this included the consensus criteria used at the time the data were gathered and SOFA-scores were not available for patients in Cohort A [22]. Infection-induced organ dysfunction was defined as a probable or verified infection based on clinical presentation, laboratory results, microbiological samples and radiologic examinations, and an acute organ dysfunction of no other apparent or pre-existing cause. For the definitions of organ dysfunction see Table 1.

The Severinghaus equation was used to calculate Partial pressure of Oxygen/Fraction of Inspired Oxygen (PaO_2_/FiO_2_) ratio for patients with oxygen saturation (SaO_2_) 90%–94% and for patients with chronic obstructive pulmonary disorder (COPD) and SaO_2_ 87%–95% and simultaneous oxygen supply in Cohort B [23].

Acute neurological dysfunction was not included as an organ dysfunction defining severe sepsis, since it was part of scores being evaluated. When evaluating the effect of adding lactate to risk stratification scores, hyperlactaemia was excluded from the definition for severe sepsis.

The different scores were primarily evaluated against a composite outcome (hereafter called severe infection) of severe sepsis (according to sepsis-2), admission to intensive care within 72 h due to an infection or infection-related mortality within 72 h.

qSOFA was defined as one point for each of respiratory rate ≥ 22, SBP ≤ 100 mm Hg and altered mental status. When calculating sensitivity and specificity, a cut-off of two points was used.

The NEWS2 definition is presented in Table S1. For the calculations of sensitivity and specificity, the score was dichotomized at a cut-off of five points [7].

When testing for the addition of lactate to qSOFA, qSOFA was revised to a four-point score with one point added for lactate > 2.0 mmol/L. A lactate level of >2.0 mmol/L was chosen as in the original assessment of qSOFA [13].

When testing for the addition of HBP to qSOFA, qSOFA was also revised to a four-point score with one point added for HBP > 30 ng/mL.

Lactate was analysed at the clinical chemistry departments at each hospital. The reference interval at the hospitals were lactate < 2.2 mmol/L. Hence the cut-off for organ dysfunction due to hyperlactaemia was set at >3.2, (>1mmol/L over the normal reference value) [24].

HBP was analysed with enzyme-linked immunosorbent assay (ELISA) at a centralized laboratory.

Definitions for comorbidities are found in Table S3.

### 2.3. Statistical Methods

Area under receiver operating characteristic curve (AUC), sensitivity, specificity and their 95% confidence interval (CI) were calculated. *p*-values for medians were calculated using a Mann–Whitney U test, *p*-values for proportions were calculated using chi^2^ and Fishers’ exact test as appropriate and the formula of DeLong was used for comparison of AUC. *p*-values below 0.05 were regarded as significant.

Patients with missing values in parameters in qSOFA and NEWS2 were excluded in the primary analysis.

Multiple imputation of missing values that are part of the risk stratification scores or missing values for biomarkers were executed using predictive mean matching and logistic regression with 20 imputation sets and the performances of different risk stratification scores were calculated in a sensitivity analysis. AUCs and 95% CIs were calculated as medians of the pooled data in the imputed data sets.

To assess the performance of NEWS2 and qSOFA in relation to the proportion of patients with organ dysfunction a sensitivity analysis was performed. Patients were divided into the presence or absence of organ dysfunction. Samples from respective groups were drawn (with replacement) to mimic cohorts with the proportion of patients with organ dysfunction ranging from 1% to 99% in steps of 1%. For each proportion of patients with organ dysfunction 100 stochastic cohorts (each with 1000 patients) were drawn and the AUC-values for qSOFA and NEWS2 were calculated and the median was calculated and plotted.

Analyses were performed using SPSS software system version 23.0 (IBM, Armonk, NY, USA) and R: A language and environment for statistical computing. (R Foundation for Statistical Computing, Vienna, Austria. URL https://www.R-project.org/) with the following packages: readxl, ggplot2, grid, gridExtra, clinfun, pROC and epiR.

## 3. Results

### 3.1. Cohort A—Patients with Suspected Infection

#### 3.1.1. Patient Demographics

**Figure 1:**
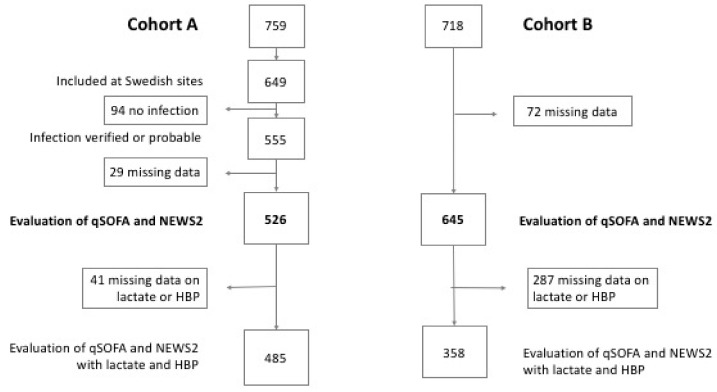
Flow chart. Cohort A, patients with suspected infection and one of systemic inflammatory response syndrome (SIRS) criteria, or self-reported fever or chills. Cohort B, patients with one of the following parameters: respiratory rate > 25, heart rate > 120, altered mental awareness, SBP < 100 mmHg, SaO_2_ < 90%, or <93% if ongoing treatment with oxygen. qSOFA (Quick Sequential Organ Failure Assessment). NEWS2 (National Early Warning Score 2). HBP (heparin binding protein).

Six-hundred and forty-nine patients were eligible for the study, 94 patients without a probable or verified infection and 29 patients with missing data were excluded. A total of 526 patients with infection were included in the primary analysis of Cohort A. The composite outcome (consisting of sepsis with organ dysfunction, admission to intensive care within 72 h due to an infection or infection-related mortality within 72 h from enrolment) were found in 238 (45%) patients (Figure 1). The 30-day mortality was 3%. Patient characteristics are presented in Table 2.

Forty-one patients included in the study, but with missing lactate or HBP-analysis were excluded when the effect of adding lactate or HBP to qSOFA and NEWS2 were studied.

#### 3.1.2. Performances of the Risk Stratification Scores for the Composite Outcome and Mortality

NEWS2 had a significantly higher AUC, 0.80 (95% CI 0.75–0.83), than qSOFA, 0.71 (95% CI 0.66–0.75), *p* < 0.01. The suggested cut-off of ≥2 qSOFA was specific, 97% (95% CI 0.95–0.99), but yielded low sensitivity, 17% (95% CI 0.12–0.22). Cross tabulations, sensitivity, specificity, AUC and Odds Ratio (OR) for qSOFA and NEWS2 can be found in Table 3.

#### 3.1.3. The Effect of Adding Biomarkers to qSOFA and NEWS2

The effect of adding lactate or HBP to qSOFA and NEWS2, respectively, was analysed in the 485 patients with biomarkers available. In these analyses organ dysfunction due to hyperlactaemia was excluded from the outcome. There was no significant improvement of adding lactate to qSOFA. With the addition of one point for lactate > 2.0 mmol/L to qSOFA, the AUC was increased from 0.70 (95% CI 0.66–0.75) to 0.73 (95% CI 0.68–0.77), *p* = 0.37.

The addition of one point for HBP > 30 ng/mL increased the AUC for qSOFA from 0.70 (95% CI 0.66–0.75) to 0.78 (95% CI 0.74–0.82), *p* = 0.01 (Table 4).

#### 3.1.4. Sensitivity Analyses—Outcome

The evaluations of the risk stratification scores were repeated with acute neurological dysfunction as organ dysfunction defining severe sepsis. This did not significantly change the results. The same was true when adding hyperlactaemia as organ dysfunction to the analyses with lactate and HBP (Table 4).

#### 3.1.5. Sensitivity Analyses—Missing Data

The analyses for the diagnostic accuracy for qSOFA and NEWS2 with and without lactate or HBP were repeated after multiple imputation of missing data. This was performed for the 29 patients excluded for missing data and for 41 patients included in the primary analysis but with missing biomarkers. For missing data, see Table S4. Data were assumed to be missing at random. Distribution of variables were similar for patients with missing values and patients with complete data. Variables imputed were systolic blood pressure, heart frequency, respiratory frequency, temperature, mental status, SaO_2_, oxygen treatment, lactate and HBP. Other parameters in the imputation although not imputed were comorbidities and outcome. The models were validated by plots of imputations and iterations.

The performances of the risk stratification scores were analysed in imputed data sets, (Table S5). The analyses using multiple imputation yielded similar estimates as the analyses of the original data.

### 3.2. Cohort B—Patients, with and without Infection

#### 3.2.1. Patient Demographics

Seventy-three patients were excluded from the study due to missing data. Of the 645 patients included in the primary analyses, 269 (42%) had a diagnosed infection. The composite outcome of sepsis with organ dysfunction was experienced in 191 (30%), i.e., admission to intensive care due to an infection or infection-related mortality within 72 h from enrolment (Figure 1). Data on patient characteristics are presented in Table 2.

Patients with a probable or proven infection had a 30-day mortality rate of 12%. Patients without an infection had a 30-day mortality rate of 9%.

Of the patients included in the study, 287 with missing lactate or HBP-analysis were excluded when the effect of adding lactate or HBP to qSOFA and NEWS2 were studied. The large number of missing analyses was because all blood samples with signs of haemolysis were discarded in the original study.

#### 3.2.2. Performance of the Risk Stratification Scores for the Composite Outcome and Mortality

Cross tabulations, sensitivity, specificity, AUC and OR for qSOFA and NEWS2 are presented in Table 3. NEWS2 had significantly higher AUC, 0.70 (95% CI 0.65–0.74), than qSOFA, AUC 0.62 (95% CI 0.57–0.67), *p* = 0.02.

#### 3.2.3. The Effect of Adding Biomarkers to qSOFA and NEWS2

The effect of adding lactate or HBP to qSOFA and NEWS2, respectively, was analysed in the 358 patients with biomarkers available. In these analyses organ dysfunction due to hyperlactaemia was excluded. There was no effect of adding lactate to qSOFA. Without and with the addition of one point for lactate > 2.0 mmol/L the AUC increased slightly from 0.62 (95% CI 0.55–0.68) to 0.64 (95% CI 0.58–0.70) although not significantly (*p* = 0.66). Neither did the addition of one point for HBP > 30 ng/mL improve qSOFA significantly, although the AUC increased from 0.62 (95% CI 0.55–0.68) to 0.66 (95% CI 0.60–0.73), *p* = 0.37 (Table 4).

#### 3.2.4. Sensitivity Analyses—Outcome

As in Cohort A, the evaluations of the risk stratification scores were repeated with acute neurological dysfunction as an organ dysfunction, without significant effect on the results. Hyperlactaemia excluded as an organ dysfunction resulted in no significant alterations of the results (Table 4).

#### 3.2.5. Sensitivity Analyses—Missing Data

The analyses for the primary outcome were repeated after multiple imputation of missing data for 62 patients were excluded for missing data, and 287 patients with missing biomarkers. For missing data, see Table S4. Data were assumed to be missing at random. Distribution of variables were similar for patients with missing values and patients with complete data. Variables imputed were systolic blood pressure, heart frequency, respiratory frequency, temperature, mental status, SaO_2_, oxygen treatment, lactate and HBP. Other parameters in the imputation although not imputed were comorbidities and outcome. The models were validated by plots of imputations and iterations.

The performances of scores were analysed in imputed data sets, see Table S5, the analyses using multiple imputation yielded similar estimates as the analyses of the original data.

#### 3.2.6. Sensitivity Analyses—Inclusion Bias

To assess the possible inclusion bias due to convenience inclusion in Cohort A and at one site in Cohort B, a sub analysis was performed. The subgroup of Cohort A that fulfilled the inclusion criteria for Cohort B and the subgroup of Cohort B that were included consecutively and fulfilled the inclusion criteria for Cohort A was compared for performances of the risk stratification scores.

The subgroup of Cohort A consisted of 190 patients of whom 129 had the composite outcome. AUC for NEWS2 was 0.79 (95% CI 0.72–0.85) and for qSOFA 0.67 (95% CI 0.59–0.75).

The subgroup of Cohort B consisted of 171 patients, 118 with the composite outcome. The AUC for NEWS2 was 0.77 (95% CI 0.70–0.84) and for qSOFA was 0.69 (95% CI 0.61–0.78).

To further assess if any inclusion bias in Cohort A could have skewed the results a sensitivity analysis was made addressing the proportion of patients with organ dysfunction in the cohort. In Cohort A the proportion of patients developing any organ dysfunction (excluding neurological dysfunction) was 46%. Patients were divided in two groups as to the presence or absence of organ dysfunction and new samples were drawn from respective groups until a new cohort of 1000 patients was created. The sampling was adjusted so that the proportion of patients with organ dysfunction varied from 1%–99% in steps of 1%. For each cohort, the AUC for the composite outcome was calculated (Figure S1). Across all proportions of patients with organ dysfunction, the AUCs for NEWS2 were superior to the AUCs for qSOFA. This generalizability of the results suggests that even if there might have been an inclusion bias possibly leading to an overrepresentation of patients with organ dysfunction the result, that NEWS2 is superior to qSOFA, would still be valid.

## 4. Discussion

In this evaluation of risk stratification scores for sepsis in the ED, NEWS2 was superior to qSOFA in screening for the composite outcome; sepsis with organ dysfunction, infection-related mortality or intensive care due to an infection. The superiority of NEWS2 compared to qSOFA was true both among infected patients (Cohort A) as well as undifferentiated patients (Cohort B).

In agreement with the finding by Seymour et al. accuracy did not improve through addition of lactate to qSOFA [4]. HBP has previously been shown to be superior to lactate in predicting sepsis [19,25] and thus the addition of HBP to qSOFA was tested. HBP improved the performance of qSOFA significantly in infected patients (Cohort A) but NEWS2 still performed better. NEWS has previously been shown to be superior to qSOFA for detection of sepsis at ED [26]. NEWS2 differs from NEWS in including different SaO_2_-scales and the addition of altered mentation, which is why the higher AUC for NEWS2 than qSOFA in this study was not surprising [7].

The higher AUCs in Cohort A for both scores, which were due to higher specificities yet lower sensitivities, is likely multifactorial. Of particular importance was that Cohort A was more homogeneous, consisting only of infected patients and also due to the differences in inclusion criteria.

Whether a study determines a risk stratification score to be of value is highly dependent upon the chosen outcome. Many prior studies regarding qSOFA have used mortality as the primary outcome [27,28]. However, focusing only on mortality implies that worsening in physiologic outcomes are not clinically important. As long-term effects of sepsis have become more apparent, the development of sepsis itself is an important outcome [29,30,31].

Compared to studies with mortality as the primary outcome, the AUC and sensitivity of qSOFA was somewhat lower in our study, however this agreed with previous studies using sepsis as the primary outcome [10,12,32,33]. Evaluating in reference to the most severely ill patients might lead to overestimation of sensitivity and negative predictive value. A recent study by Usman et al. used severe sepsis as outcome but with higher AUC for qSOFA than in the present study. The inclusion of central nervous dysfunction in the outcome as well as the shorter period evaluated for sepsis probably contributed to the higher AUC; for several patients in our study sepsis was detected after eight hours [21,26]. Although we found very high specificity, the poor sensitivity of two qSOFA points diminishes its usefulness in an ED setting when screening for a condition like sepsis, which aims to catch all potential patients for rapid treatment [3]. We included 30-day mortality as a secondary outcome. The mortality in both cohorts was low, generating wide confidence intervals for 30-day mortality.

Strengths of the study included an independent physician review of whether infection and organ dysfunction were present, use of a composite outcome i.e., sepsis with organ dysfunction, infection-related mortality or intensive care due to an infection provides a “real world utility”. This composite score provides a more global view of patient outcome than the in-hospital mortality [4,32,34,35]. The weakness of the composite score was that several parameters in the risk stratification scores also define organ dysfunction and thus sepsis. Hence, risk stratification scores with these sepsis-defining parameters can be misleading when evaluated against sepsis. Unlike other studies, we tried to minimize this bias by excluding organ dysfunction of the central nervous system from the outcome. Furthermore, when evaluating the effect of adding lactate to the risk stratification scores, hyperlactaemia was excluded from the sepsis definition [11,26,33]. To further elaborate on this, we repeated the analyses with acute neurological dysfunction as well as hyperlactaemia included as organ dysfunctions with no significant alterations in results. These findings illustrated the robustness of our analysis.

One might fear that also other parameters such as vital signs which were present in the scores as well in the definition of sepsis might skew the results. However, this bias was inevitable since there is no gold standard diagnostic test for sepsis against which different scores can be compared. One method to probe this bias would be to instead address the predictive validity, which evaluates the relative performance of the score by their ability to identify those patients at increased risk for downstream events associated with the condition of interest. In the context of this, attributable mortality (adjusted for known risk factors) could have been performed. However, we felt this approach did not significantly reduce the bias.

A weakness of the study was the inclusion criteria in Cohort B. Some of the lower limits for inclusion criteria are in fact above those needed for points to accrue in the scoring systems, which may have affected the results. For example, in Cohort B, patients with a respiratory rate below 25 might have been excluded. This group may contain patients with all kinds of classifications according to the scores and outcome and we could not properly foresee how it affected the performances of the scores. Furthermore, these criteria for inclusion were also likely to favour inclusion of more severely ill patients than the inclusion of all ED patients thus giving a higher mortality in Cohort B compared to other ED cohorts where all patients with ED encounters were included. However, the threshold for seeking the ED varies depending on the location and whether a referral or not is mandatory or if the ED is the primary site where people seek medical advice for illnesses and hence we felt it was necessary to establish predefined inclusion criteria. Many other studies have instead only included patients admitted to the hospital but since admittance criteria differ between hospitals and countries we preferred to use predefined inclusion criteria based on vital parameters.

Another weakness was the potential selection bias caused by convenience inclusion of patients in Cohort A and at one site in Cohort B. There may have been a risk that patients with sepsis were more often included than patients without sepsis and that one scoring system might have been favoured in front of the other. To address this a sensitivity analyses compared the sub cohort of patients that were consecutively included with convenience-included patients from the Cohort A. The sensitivity analysis rendered similar performances of the risk stratification scores. This suggests that inclusion bias was unlikely. However, this analysis did not completely exclude a selection bias of patients in Cohort A not fulfilling the inclusion criteria of Cohort B. To address this possibility another sensitivity analysis was made. Statistically simulated cohorts were created where the proportion of patients with organ dysfunction varied between 1% and 99% and across the range of frequencies NEWS2 had a higher AUC than qSOFA. This sensitivity analysis demonstrated a robustness in the results and indicated that even if there might have been an inclusion bias possibly leading to overrepresentation of patients with organ dysfunction, the superiority of NEWS2 versus qSOFA would still be valid in cohorts with lower frequencies of patients with organ dysfunctions.

qSOFA was proposed to identify those who are more likely to have a poor outcome among patients with a suspected infection [4]. It was also suggested that patient with ≥2 qSOFA should prompt consideration of infection [13]. Furthermore, the rationale for suspecting infection is highly variable. For example, in Cohort B, 33% of those seeking with infection had no infection, while 34% of those found later to have an infection, had a chief complaint unrelated to infection. This lack of initial diagnostic precision is why we wanted to evaluate the qSOFA, NEWS2 and biomarkers in both a cohort of only infected patients as well as in a cohort of undifferentiated patients.

## 5. Conclusions

To the best of our knowledge, NEWS2 has not been evaluated for its ability to predict sepsis in patients presenting to the ED, nor has it been compared with qSOFA for this purpose. In this study we compared the ability of NEWS2 and qSOFA to predict sepsis both among infected patients and among undifferentiated patients in the ED. We found that NEWS2 was superior to qSOFA for detecting sepsis with organ dysfunction, admission to intensive care due to an infection and infection-related mortality.

## Figures and Tables

**Table 1:** Organ dysfunction definition.

System	Values
Cardiovascular	SBP < 90 mmHg ∆SBP > −40 MAP < 70 Vasopressor
Respiratory, Cohort A	SaO_2_ < 90%
Respiratory, Cohort B	SaO_2_ < 90% For COPD SaO_2_ < 87% For SaO_2_ 90%–94% and O_2_ PaO_2_/FiO_2_ ratio < 300 with COPD and SaO_2_ 87%–95% PaO_2_/FiO_2_ ratio < 250
Renal	creatinine increase > 44 µmol/L urinary output < 0.5mL/kg/h for >2 h initiation of dialysis
Hepatic	bilirubin > 35 μmol/L
Haematologic	platelet count < 100 × 10^9^/L INR > 1.5 unless use of anticoagulants
Metabolic	lactate > 3.2 mmol/L

Systolic blood pressure (SBP), mean arterial blood pressure (MAP), oxygen saturation (SaO_2_), chronic obstructive pulmonary disease (COPD), international normalized ratio (INR).

**Table 2:** Patient characteristics. Percentages within brackets.

	Cohort A, *n* = 526			Cohort B, *n* = 645		
	With Composite Outcome *n* = 238	Without Composite Outcome *n* = 288	*p*	With Composite Outcome *n* = 191	Without Composite Outcome *n* = 454	*p*
**Age, median**	70	52	<0.01	76	71	<0.01
**Female, *n* (%)**	109 (46)	125 (43)	0.60	94 (49)	218 (48)	0.80
**Comorbidities *n* (%)**						
**Diabetes**	48 (20)	30 (10)	<0.01	43 (23)	80 (18)	0.16
**Cardiovascular disease**	80 (34)	34 (12)	<0.01	99 (52)	205 (45)	0.14
**Renal disease**	16 (6)	32 (13)	<0.01	56 (29)	111 (24)	0.20
**Liver disease**	1 (0)	3 (1)	0.63	12 (6)	13 (3)	0.05
**Malignancy**	24 (10)	21 (7)	0.28	31 (16)	57 (13)	0.21
**Immunodeficiency**	9 (4)	10 (4)	1	8 (4)	6 (1)	0.04
**Respiratory disease**	32 (13)	22 (8)	0.03	32 (17)	50 (11)	0.05
**No comorbidities**	98 (41)	195 (68)	<0.01	31 (16)	148 (33)	<0.01
**Sepsis-2 organ dysfunction, *n* (%)**						
**No organ dysfunction**	5 (2)	281 (98)	<0.01	0 (0)	212 (47)	<0.01
**Neurologic**	30 (13)	7 (2)	<0.01	52 (27)	91 (20)	0.05
**Cardiovascular**	174 (91)	-	-	126 (66)	130 (29)	<0.01
**Respiratory**	63 (27)	-	-	112 (59)	83 (18)	<0.01
**Renal**	28 (12)	-	-	50 (26)	33 (7)	<0.01
**Haematological**	25 (11)	-	-	19 (10)	26 (6)	0.05
**Hepatic**	9 (4)	-	-	7 (4)	6 (1)	0.06
**Intensive Care, *n* (%)**	14 (6)	-	-	18 (10)	30 (7)	0.25
**Three-days mortality, *n* (%)**	3 (1)	-	-	7 (4)	18 (4)	0.86

**Table 3:** Accuracy of risk stratification scores.

	qSOFA		NEWS2	
	≥2	<2	≥ 5	<5
**Cohort A (*n* = 526)**				
**Composite outcome +**	40	198	155	83
**Composite outcome –**	8	280	57	231
**Sensitivity**	0.17 (0.12–0.22)		0.65 (0.59–0.71)	
**Specificity**	0.97 (0.95–0.99)		0.80 (0.75–0.85)	
**AUC**	0.71 (0.66–0.75)		0.80 (0.75–0.83)	
***p* compared to qSOFA**	reference		<0.01	
**30-days mortality +**	6	7	9	4
**30-days mortality –**	42	471	203	310
**AUC 30-days mortality**	0.72 (0.54–0.90)		0.75 (0.60–0.90)	
**Cohort B (*n* = 645)**				
**Composite outcome +**	67	124	160	31
**Composite outcome –**	75	379	287	167
**Sensitivity**	0.35 (0.28–0.42)		0.84 (0.78–0.89)	
**Specificity**	0.83 (0.80–0.87)		0.37 (0.32–0.41)	
**AUC**	0.62 (0.57–0.67)		0.70 (0.65–0.74)	
***p* compared to qSOFA**	reference		0.02	
**30-days mortality +**	18	32	45	5
**30-days mortality –**	79	338	289	128
**AUC 30-days mortality**	0.62 (0.53–0.70)		0.70 (0.63–0.70)	

95% CI within brackets. AUC (area under receiver operating characteristic curve). qSOFA (Quick Sequential Organ Failure Assessment). NEWS2 (National Early Warning Score 2).

**Table 4:** Accuracy of risk stratification scores with biomarkers. 95% CI within brackets. Organ dysfunction (OD).

	qSOFA	qSOFA Incl Lactate	qSOFA Incl HBP	NEWS2	NEWS2 Incl Lactate	NEWS2 Incl HBP
**Cohort A, *n* = 485**						
**Composite outcome excl lactate and CNS OD**						
**AUC**	0.70 (0.66–0.75)	0.73 (0.68–0.77)	0.78 (0.74–0.82)	0.79 (0.75–0.83)	0.80 (0.76–0.84)	0.81 (0.78–0.85)
***p* compared to qSOFA**	reference	0.37	0.01	<0.01	<0.01	<0.01
***p* compared to NEWS2**	<0.01	0.06	0.74	reference	0.73	0.73
**Composite outcome incl lactate excl CNS OD**						
**AUC**	0.71 (0.66–0.75)	0.74 (0.70–0.78)	0.78 (0.74–0.82)	0.79 (0.75–0.83)	0.80 (0.76–0.84)	0.82 (0.78–0.85)
***p* compared to qSOFA**	reference	0.47	0.03	0.01	<0.01	<0.01
***p* compared to NEWS2**	0.01	0.11	0.73	reference	0.73	0.30
**Composite outcome incl lactate and CNS OD**						
**AUC**	0.72 (0.68–0.77)	0.76 (0.71–0.80)	0.80 (0.76–0.84)	0.81 (0.77–0.85)	0.82 (0.78–0.86)	0.83 (0.80–0.87)
***p* compared to qSOFA**	reference	0.21	0.01	<0.01	<0.01	<0.01
***p* compared to NEWS2**	<0.01	0.09	0.73	reference	0.72	0.47
**Cohort B, *n* = 358**						
**Composite outcome excl lactate and CNS OD**						
**AUC**	0.62 (0.55–0.68)	0.64 (0.58–0.70)	0.66 (0.60–0.73)	0.67 (0.61–0.73)	0.68 (0.62–0.73)	0.69 (0.63–0.75)
***p* compared to qSOFA**	reference	0.66	0.37	0.26	0.18	0.11
***p* compared to NEWS2**	0.26	0.50	0.82	reference	0.82	0.65
**Composite outcome incl lactate excl CNS OD**						
**AUC**	0.62 (0.55–0.68)	0.64 (0.58–0.70)	0.66 (0.60–0.73)	0.67 (0.61–0.73)	0.68 (0.62–0.73)	0.69 (0.63–0.75)
***p* compared to qSOFA**	reference	0.66	0.37	0.26	0.18	0.11
***p* compared to NEWS2**	0.26	0.50	0.82	reference	0.82	0.65
**Composite outcome incl lactate and CNS OD**						
**AUC**	0.62 (0.56–0.68)	0.64 (57–0.70)	0.67 (0.61–0.73)	0.68 (0.62–0.74)	0.68 (0.62–0.74)	0.69 (0.63–0.75)
***p* compared to qSOFA**	reference	0.66	0.26	0.18	0.18	0.11
***p* compared to NEWS2**	0.18	0.36	0.82	reference	1	0.82

95% CI within brackets. qSOFA (quick Sequential Organ Failure Assessment). NEWS2 (National Early Warning Score 2). OD (organ dysfunction). CNS (central nervous system). HBP (heparin binding protein).

## Supplementary Materials

The following are available online at https://www.mdpi.com/2077-0383/8/8/1128/s1, Table S1: National Early Warning Score 2 (NEWS2). Table S2: Periods of inclusion. Table S3: Definitions for comorbidities. Table S4: Missing data. Table S5: AUC for different risk stratification scores, missing values substituted by multiple imputation. Figure S1: The performance of NEWS2 and qSOFA change with the frequency of organ dysfunction in the cohort.
