# Supplementary material for: NEWS2 Is Superior to qSOFA in Detecting Sepsis with Organ Dysfunction in the Emergency Department
Source: J Clin Med. 2019 Jul 29;8(8):1128. doi: 10.3390/jcm8081128 (PMC6723972; doi:10.3390/jcm8081128)
Supplement: Supplementary file 1 [file jcm-08-01128-s001.pdf]

Supplementary Material

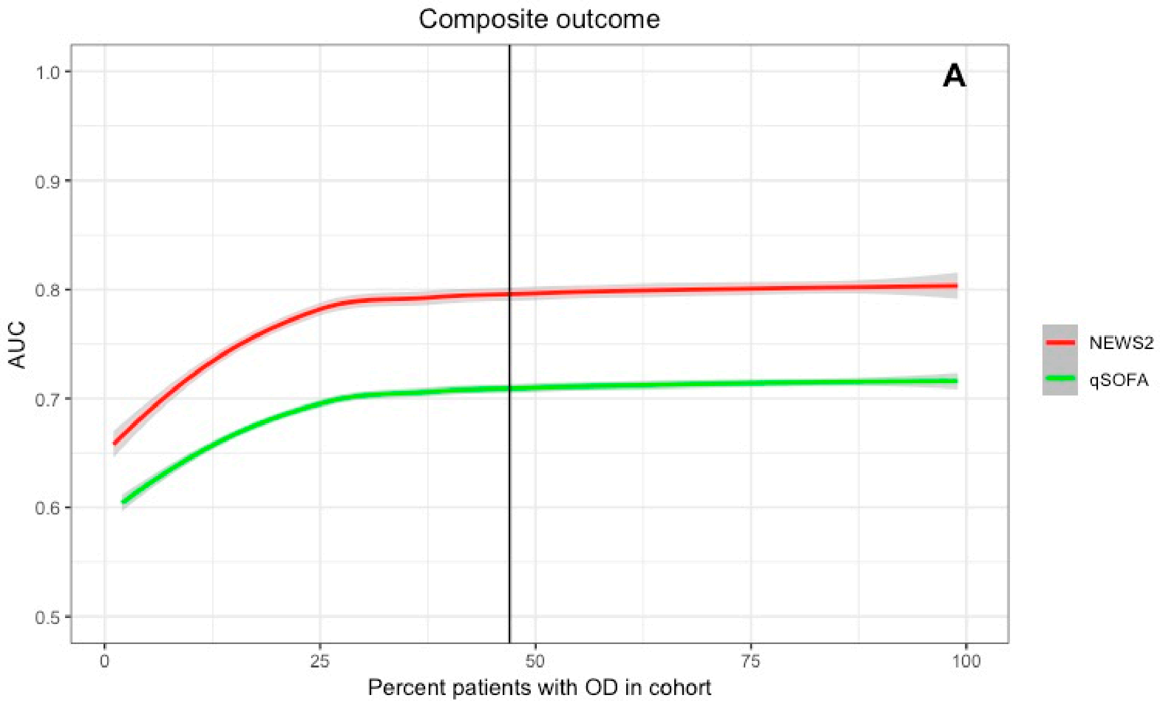

Figure 1. The performance of NEWS2 and qSOFA change with the frequency of organ dysfunction in the cohort.

Patients were divided in two groups as to the presence or absence of organ dysfunction (excluding neurological dysfunction). Samples from the two groups were drawn to mimic cohorts with the proportion of patients with organ dysfunction ranging from 1% to 99% in steps of 1%. For each proportion of patients with organ dysfunction 100 stochastic cohorts were drawn with replacement and the AUC-values for NEWS2 and qSOFA were calculated and the median was calculated and plotted. The vertical line represents the proportion of patients with organ dysfunction in the original whole cohort.

Table 1. National Early Warning Score 2 (NEWS2).

	3	2	1	0	1	2	3
Respiration rate	<9		9–11	12-20		21–24	>24
SpO_2 Scale 1	<92	92–93	94–95	>95			
SpO_2 Scale 1	<84	84–85	86-87	88–92 >92 on air	93–94 on oxygen	95–96 on oxygen	>96 on oxygen
Air or Oxygen		Oxygen		Air			
SBP	<91	91–100	101–110	111–219			>219
Pulse	<41		41–50	51–90	91–110	111–130	>130
Consciousness				Alert			VPCU
Temperature	<35.1		35.1–36.0	36.1–38.0	38.1–39.0	>39.0	

Table 2. Periods of inclusion.

Site	Catchment area	Period of inclusion	Days with inclusion
Cohort A			
Lund	334 000	2011-03-30 – 2011-11-01	117
Örebro[1]	180 000	2011-04-24 – 2011-12-07	87
Malmö	420 000	2011-03-12 – 2011-11-11	67
Linköping	150 000	2011-05-17 – 2011-09-14	38
Cohort B			
Lund	334 000	2015-02-02 – 2015-02-27 + 2016-01-21 – 2016-03-18	62
Bern[2]	1 500 000	2015-02-18 – 2015-04-10	33
Vancouver[2]	650 000	2015-03-11 – 2015-04-10	23
Helsingborg	250 000	2015-04-13 – 2016-01-21	84

[1] only at ED for infectious diseases, [2]more hospitals in the catchment area.

Table 3. Definitions for comorbidities.

**Diabetes**	- **All patients with a diabetes diagnosis**
Cardiovascular Disease	- Ischemic heart disease including previous myocardial infarction or present medication for angina pectoris<br>- Chronic heart failure requiring continuous medication<br>- Peripheral vascular disease including claudicatio intermittens
Renal Disease	- Proof of elevated serum creatinine registered as chronic in medical history<br>- Chronic kidney replacement therapy<br>- Chronic renal failure.<br>- Haemodialysis or chronic peritoneal dialysis
Respiratory Disease	- Chronic obstructive or restrictive disease that requires daily medication or results in limitations in daily life<br>- Diagnosis of pulmonary hypertension<br>- Ongoing medication for other lung diseases (e.g. sarcoidosis, lung fibrosis, tuberculosis)<br>- Asthma bronchiale requiring regular medication
Liver Disease	- Cirrhosis score with a positive biopsy and documented portal hypertension,<br>- Previous episodes with high gastro-intestinal bleeding due to portal hypertension,<br>- Previous episodes with liver failure, coma or encephalopathy<br>- Other chronic hepatic disease
Malignancy	- Proven by imaging, biopsy, surgery or by tumour markers
Immunodeficiency	- Ongoing (or present) immunosuppressive therapy, chemotherapy, or radiotherapy<br>- Haematological malignancy such as leukaemia, lymphoma and myeloma<br>- HIV/AIDS

Table S4. Missing data.

	Cohort A *n* = 555		Cohort B *n* = 707	
	n	%	n	%
**Systolic Blood Pressure**	4	<1	6	<1
**Heart Frequency**	1	<1	3	<1
**SaO2**	10	2	24	3
**Respiratory Frequency**	7	1	17	2
**Temperature**	8	1	23	3
**Mental Status**	6	1	5	<1
**Lactate**	32	6	232	33
**HBP**	18	3	178	25
**Oxygen treatment**	6	1	24	3

Table 5. AUC for different risk stratification scores for sepsis with organ dysfunction (excl organ dysfunction in central nervous system or hyperlactemia), admission to intensive care within 72 hours due to an infection or infection-related mortality within 72 hours from enrolment, missing values were substituted by multiple imputation.

	qSOFA	qSOFA incl lactate	qSOFA incl HBP	NEWS2	NEWS2 incl lactate	NEWS2 incl HBP
			Cohort A			
AUC	0.71	0.73	0.79	0.79	0.80	0.82
95% CI	0.67–0.75	0.69–0.77	0.75–0.83	0.76–0.83	0.76–0.84	0.78–0.85
p compared to qSOFA	reference	0.52	0.01	0.01	<0.01	<0.01
p compared to NEWS2	0.01	0.04	1	reference	0.72	0.27
			Cohort B			
AUC	0.63	0.63	0.67	0.70	0.70	0.71
95% CI	0.58–0.67	0.58–0.67	0.63–0.72	0.66–0.74	0.66–0.74	0.67–0.76
p compared to qSOFA	reference	1	0.23	0.03	0.03	0.01
p compared to NEWS2	0.03	0.03	0.35	reference	1	0.75
